# Sustained release of brimonidine from BRI@SR@TPU implant for treatment of glaucoma

**DOI:** 10.1080/10717544.2022.2039806

**Published:** 2022-02-17

**Authors:** Yujin Zhao, Chang Huang, Zhutian Zhang, Jiaxu Hong, Jianjiang Xu, Xinghuai Sun, Jianguo Sun

**Affiliations:** a Eye Institute and Department of Ophthalmology & Visual Science, Eye & ENT Hospital, Shanghai Medical College, Fudan University, Shanghai, China; b NHC Key Laboratory of Myopia (Fudan University), Key Laboratory of Myopia, Chinese Academy of Medical Sciences, Shanghai Key Laboratory of Visual Impairment and Restoration, Shanghai, China; c State Key Laboratory of Medical Neurobiology and MOE Frontiers Center for Brain Science, Institutes of Brain Science, Fudan University, Shanghai, China

**Keywords:** Sustained release, brimonidine, intraocular pressure (IOP), glaucoma, conjunctival sac implant

## Abstract

Glaucoma is the leading cause of irreversible vision loss worldwide, and reduction of intraocular pressure (IOP) is the only factor that can be interfered to delay disease progression. As the first line and preferred method to treat glaucoma, eye drops have many shortcomings, such as low bioavailability, poor patient compliance, and unsustainable therapeutic effect. In this study, a highly efficient brimonidine (BRI) silicone rubber implant (BRI@SR@TPU implant) has been designed, prepared, characterized, and administrated for sustained relief of IOP to treat glaucoma. The *in vitro* BRI release from BRI@SR@TPU implants shows a sustainable release profile for up to 35 d, with decreased burst release and increased immediate drug concentration. The carrier materials are not cytotoxic to human corneal epithelial cells and conjunctival epithelial cells, and show good biocompatibility, which can be safely administrated into rabbit’s conjunctival sac. The BRI@SR@TPU implant sustainably released BRI and effectively reduced IOP for 18 d (72 times) compared to the commercial BRI eye drops (6 h). The BRI@SR@TPU implant is thus a promising noninvasive platform product for long-term IOP-reducing in patients with glaucoma and ocular hypertension.

## 1. Introduction

Glaucoma is the leading cause of irreversible vision loss worldwide, characterized by elevated intraocular pressure (IOP) and progressive optic nerve damage and visual field defects (Li et al., 2016). The number of glaucoma patients will increase to 111 million until 2040 (Tham et al., 2014). Glaucoma is commonly known as a multifactorial disease, and IOP-lowering is the only factor that can be interfered to delay disease progression (Nordstrom et al., 2005). The treatments for glaucoma mainly rely on medical therapy, especially eye drops, as the first line and preferred method. Many kinds of novel formulations have also been developed to improve the medical therapy (Sun et al., 2017; Lai et al., 2020; Luo et al., 2021). Brimonidine (BRI) is an IOP-lowing agent as the third generation α2 adrenoceptor agonist to benefit IOP relief by reducing aqueous humor production and increasing uveoscleral outflow (Adkins & Balfour, 1998). BRI eye drops are commonly administrated for IOP-lowering treatment. However, the IOP-lowing effect after applying BRI eye drops only lasts for a few hours due to its low bioavailability through the cornea (1–7%) (Ghate & Edelhauser, 2008), and daily multiple administration is required to maintain effective drug concentration and IOP-lowing treatment effect. Glaucoma is a chronic disease, thus long-term therapy and a rigorous administration schedule of eye drops are necessary. Much drug toxicity accumulated in ocular tissues will cause a poor patient compliance in the lifelong glaucoma therapy, and only 31–67% patients can adhere to the use of eye drops for 12 months (Reardon et al., 2011; Aref, 2017). Thus, it is urgently necessary to develop new types of formulations to increase drug bioavailability, improve patient compliance, and sustain therapeutic effect on glaucoma (Jain et al., 2020; Li et al., 2020; Nguyen et al., 2020; Nguyen & Lai, 2020).

**Figure 1.**
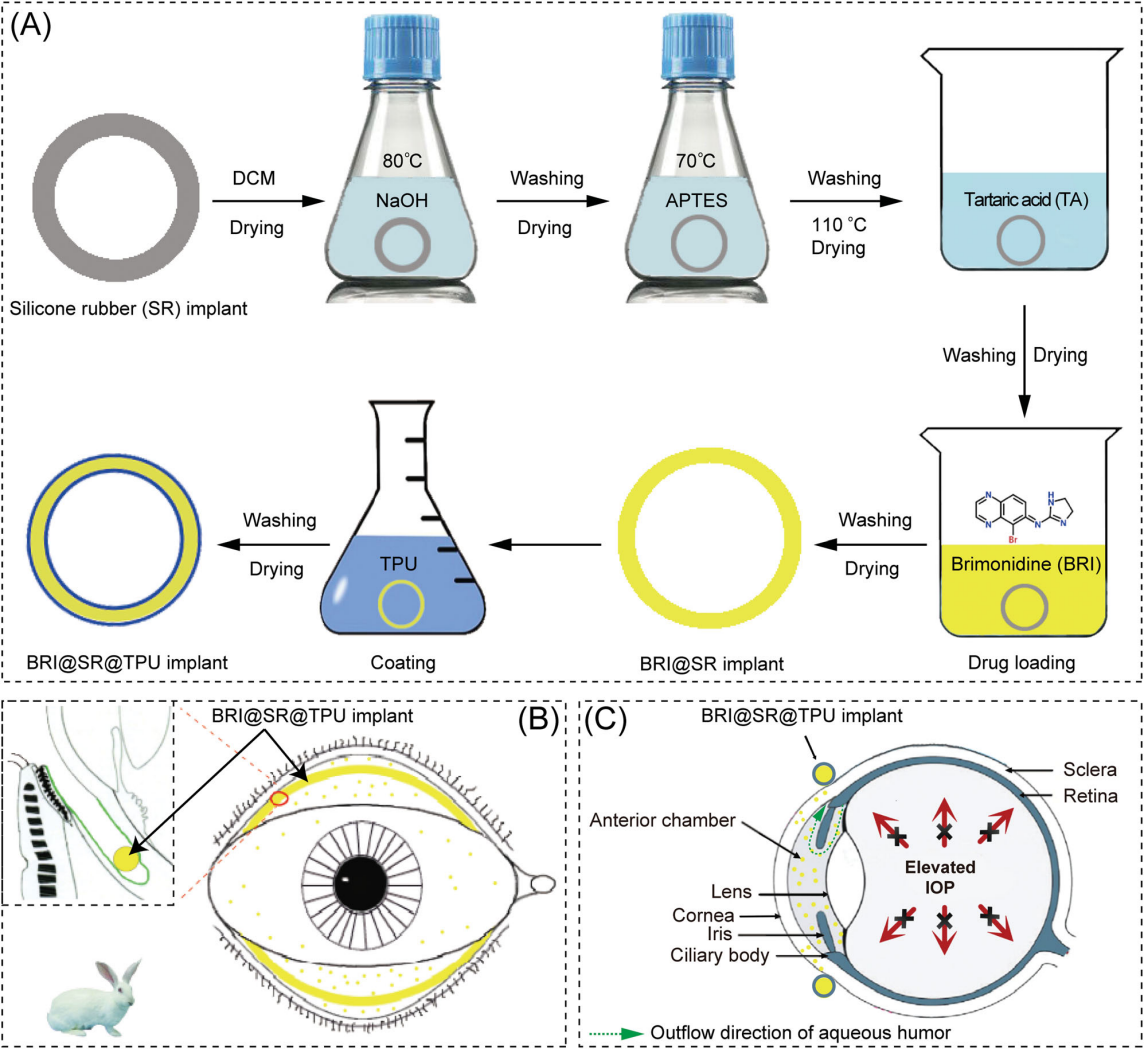
Schematic representation of the preparation courses (A), administration mode (B), and pharmacological action (C) of the BRI@SR@TPU implant. DCM: dichloromethane; APTES: γ-aminopropyl triethoxy silane; TPUs: thermoplastic polyurethanes.

BRI has been delivered for glaucoma treatment based on many kinds of drug delivery systems (DDSs) constructed by microspheres (Chiang et al., 2016), hydrogels (Cho et al., 2016), implants (Mealy et al., 2014; Ravindran et al., 2014), niosomes (Eldeeb et al., 2019), and many nanoparticles including charged nanoparticles (Ibrahim et al., 2015), lipid nanoparticles (El-Salamouni et al., 2015), chitosan nanoparticles (Singh & Shinde, 2011), albumin nanoparticles (Kim et al., 2015), poly(acrylic acid) nanoparticles (De et al., 2003), and poly(lactic acid-co-glycolic acid) (PLGA) nanoparticles (Yang et al., 2012), etc. However, in the ocular drug administration, convenience is a very important factor to improve patient compliance. Silicone rubber (SR), also known as polydimethylsiloxane, has attracted particular attention as a suitable biomaterial for preparing novel DDSs, due to its inert, biocompatible, and significant adsorption characteristics (Liu et al., 2021). The first DDS based on SR tubes was reported in 1962 by Folkman, which allowed lipophilic small molecule drug (Mw <500 Da) to diffuse through the tube-wall for prolonged drug therapy (Folkman & Long, 1964). Then, steroid hormones, antibiotics, atropine, and histamine were also reported to be loaded and released successfully by SR materials (Fenton et al., 2018). More commonly, SR materials have been used to prepare vaginal ring products (Estring^®^, Femring^®^, Progering^®^, Fertiring^®^, Annovera^®^, and Dapivirine Ring), which offer sustained or controlled delivery of therapeutic agents (Malcolm et al., 2016). Moreover, SR has also been proven to be a kind of excellent biomaterials as ophthalmic implants (Zheng et al., 2018), scleral buckling (Nguyen et al., 2001), and drug delivery carrier (Lin et al., 2019). Specifically, SR-based contact lens (CL) has been investigated to deliver several hypotensive drugs, such as timolol, betaxolol, epinephrine, and latanoprost for anti-glaucoma treatment (Musgrave & Fang, 2019). However, the majority of CL wearers suffer from significant ocular discomforts or complications, such as dry eyes, keratitis, conjunctivitis, corneal epithelial injury, and blurred vision caused from the protein adhesion onto CL surfaces (Alvarez-Rivera et al., 2018). Thus, it is suspected that a ring-shaped SR DDS can not only remain the advantages of SR-based CL DDSs, but also overcome their shortcomings, such as corneal epithelial injury and blurred vision, due to its less sensitive residence site, conjunctival sac (Bertens et al., 2021). Based on the same consideration, the bimatoprost ocular insert has been developed by Forsight Vision5 (Menlo Park, CA), which is a SR-based DDS to load bimatoprost in the silicone matrix supported by an inner polypropylene ring (Adams et al., 2019). The bimatoprost ocular insert has performed a phase II controlled study, and is expected to become the first sustained release intraocular device to lower IOP in the primary open angle glaucoma (POAG) and ocular hypertension (OA) (Brandt et al., 2016; Rubiao et al., 2021). The aim of this research was to develop a BRI@SR@TPU implant by integrating BRI into a surface-modified SR ring which was capable of sustainably releasing BRI for IOP reduction, schematically represented in Figure 1. The BRI@SR@TPU implants were administrated between upper and lower fornices where BRI was continuously released and diffused into anterior chamber through the cornea. The *in vivo* biosafety and sustained IOP-lowering effectiveness of this BRI@SR@TPU implant were investigated in the rabbit eye.

## 2. Materials and methods

### 2.1. Materials

Brimonidine (98%) was purchased from J&K Scientific Ltd. (Shanghai, China). SR implants were purchased from Kangda Rubber (Shanghai, China). Thermoplastic polyurethane (TPU) was purchased from Lubrizol Lifescience (Bethlehem, PA). Tartaric acid, 1-(3-dimethylaminopropyl)-3-ethylcarbodiimide hydrochloride (EDC), N-hydroxysuccinimide (NHS), Dulbecco’s modified Eagle media (DMEM/F12) media and fetal bovine serum (FBS) were all purchased from Sigma-Aldrich (Shanghai, China). Sodium hydroxide (NaOH), dichloromethane, tetrahydrofuran, and anhydrous ethanol were purchased from Shanghai Lingfeng (Shanghai, China). The γ-aminopropyl triethoxy silane (APTES) was purchased from Aladdin (Shanghai, China). Diazepam injection (Shanghai Xudong Haipu Pharmaceutical Co., Ltd., Shanghai, China), xylazine hydrochloride (Jilin TAT, Jilin, China), oxybuprocaine hydrochloride eye drops (Santen, Shanghai, China), BRI eye drops (0.15%, Allergan, Madison, NJ), ofloxacin eye ointment (Shenyang Sinqi, Shenyang, China), phosphate-buffered saline (Thermo Scientific, Waltham, MA) were purchased and used as received.

### 2.2. Preparation and characterization of BRI@SR@TPU implants

#### 2.2.1. Preparation of BRI@SR@TPU implants

##### 2.2.1.1. Preparation of modified SR implants

The SR implants were washed with a large amount of dichloromethane for three times to remove unreacted small molecules and dried under a vacuum for 48 h. Then, the SR implants were modified to optimize the internal microporous structure and surface properties by the reported methods (Xiang et al., 2012). Briefly, the SR implants were added into NaOH solution (4 M) and stirred at 80 °C for 4 h. After washed with 50% ethanol aqueous solution for three times and dried under a vacuum for 24 h, the SR implants were modified in 0.1% APTES anhydrous alcohol solution at 70 °C for 3 h. The SR implants were washed in a large amount of anhydrous alcohol and then dried at 110 °C for 3 h, followed by vacuum drying for 24 h. Finally, under the catalysis of EDC and NHS, the SR implants were further modified by tartaric acid (2.5 mM in anhydrous alcohol) at room temperature (25 °C) for 24 h, followed by washing in a large amount of anhydrous alcohol and vacuum drying for 24 h.

##### 2.2.1.2. BRI loading into SR implants

Brimonidine was loaded into the SR implants by soaking method (Yan et al., 2020). Briefly, the modified SR implants were immersed in the BRI dichloromethane solution (10 mg/mL) for 10 min and then dried by solvent evaporation in a fume hood for 30 min. The above soaking and drying courses were repeated for three times. The BRI loaded SR implants were then dried in a fume hood for 24 h and under a vacuum for another 24 h, to form the BRI@SR implants.

##### 2.2.1.3. Surface coating on BRI@SR implants

TPU was coated on the surface of the BRI@SR implants to control BRI release. The TPU coating was performed by 4 or 8-time repeats of the coating course in which the BRI@SR implants were quickly immersed in the 5% TPU tetrahydrofuran solution and dried by solvent evaporation in a fume hood for 1 h. All BRI@SR implants with the TPU coating, referred as BRI@SR@TPU implants, were dried under vacuum for 24 h to completely remove organic reagents. The BRI@SR@TPU implants used in cell and animal experiments were sterilized under UV light (254 nm) for 30 minutes.

#### 2.2.2. Characterization of BRI@SR@TPU implants

The surface and interface morphologies of the SR implants, modified SR implants, BRI@SR implants, and BRI@SR@TPU implants were observed by an optical microscopy (Zeiss Axio Observer, Oberkochen, Germany) and SEM (Zeiss Sigma 300, Oberkochen, Germany). A 0.5-mm-thick cross-section of SR implants was cut for the observation of interface morphology. After coated by a 10-nm layer of gold, all implant samples were observed using SEM and the representative images were then taken. Based on the obtained SEM images, the average cross-section diameter of the implant samples was determined by randomly measuring at least 50 individual cross-section using the software of ImageJ (Bethesda, MD) and calculating their average value. Surface modification of SR implants and BRI loading into the SR implants were investigated by a Fourier transform infrared (FTIR) spectrophotometer (model 22, Bruker, Coventry, UK) which was performed in an attenuated total reflection mode and the scanning range of 4000–600 cm^−1^. The residual volatile substance and the thermal stability of SR implants, modified SR implants, BRI@SR implants, and BRI@SR@TPU implants were further investigated by thermogravimetric and differential scanning calorimeter (TG-DSC, Perkin Elmer, Waltham, MA), in which TG, derivative thermogravimetry (DTG), and DSC analyses were performed. The apparatus was calibrated with the purified indium (99.9%). Samples (5 mg) were placed in flat-bottomed aluminum pan and heated at a constant rate of 10 °C/min in an atmosphere of nitrogen in a temperature range of 30–800 °C. Tensile test was operated on a Universal Testing Machine (Instron Ernst Brinck, Canton, MA) at room temperature (25 °C) with a tensile rate of 500 mm/min. Four samples were tested under the same condition. The extensometer grips were set to 15 mm. Force and elongation measurements were recorded electronically and the resulting stress–strain tensile curves were determined.

### 2.3. *In vitro* BRI release from BRI@SR@TPU implants

The *in vitro* drug release profiles of BRI@SR implants (unmodified) and BRI@SR@TPU implants were investigated as following. A sample was put into a glass bottle containing 10 mL PBS and shaken at 37 °C and a speed of 50 rpm in the DKZ-3B shaker (Shanghai Yiheng, Shanghai, China). At 0, 1, 4, and 8 h, and 1, 2, 4, 8, 14, 21, and 35 d, an aliquot of leaching liquor (2 mL) was withdrawn, and another 2 mL fresh PBS was replenished into the release medium. The withdrawn samples were stored at −80 °C until measurement. All samples were filtered through 0.45 μm membrane and measured at 246 nm using UV-Vis spectrophotometer (NanoDrop 2000, Thermo Scientific, Waltham, MA). Each result was obtained by calculating the average value from four replicates (*n* = 4).

### 2.4. *In vitro* cytotoxicity of BRI@SR@TPU implants

The consent for experiments on human tissues was obtained from the eye banks (no. 0710020190002, Eye & ENT Hospital of Fudan University, Shanghai, China), and all procedures adhered to the tenets of the Declaration of Helsinki. Human corneal epithelial cells (HCECs) and human conjunctival epithelial cells (HConEpi) were purchased from the American Type Culture Collection (ATCC, Manassas, VA). The leaching liquor was obtained by incubating the BRI@SR@TPU implants with sterilized DMEM medium at 37 °C for 24 h, according to the recommended International Standard for Biological Testing of Medical Devices (1 g materials/5 mL extraction liquid) (Tsukimura et al., 2009). The *in vitro* cytotoxicity of BRI@SR@TPU implants was investigated by culturing HCECs or HConEpi using culture medium containing the leaching liquor of the BRI@SR@TPU implants. The cells were seeded in 96-well plates at a density of 10,000 cells/well and cultured for 24 h. After the leaching liquor of BRI@SR@TPU implants was added to the culture medium, the HCECs and HConEpi were allowed to grow for another 48 h until cell counting kit-8 (CCK-8) assay (BestBio, Shanghai, China). After the leaching liquor was removed from the 96-well plates, the diluted solution of CCK-8 (10% in DMEM, 10 μL) was added and the 96-well plate was incubated for another 2–3 h at 37 °C in 5% (v/v) CO_2_. The cell viability was investigated by detecting the absorbance of each well at 450 nm by a micro plate reader (Synergy H1 Hybrid Reader, BioTek, Winooski, VT). The culture medium was set as the control. The *in vitro* cytotoxicity of the BRI@SR@TPU implants (*n* = 4) was assessed by comparing the cell viability in experiment wells with that in the control (set as 100%).

### 2.5. IOP-lowing effectiveness of BRI@SR@TPU implants

#### 2.5.1. *In vivo* drug release

Twelve New Zealand rabbits were randomly divided into two groups (*n* = 6, male): BRI@SR@TPU implants and BRI eye drops (0.15%). After administration of BRI@SR@TPU implant or BRI eye drops, an aliquot (100 μL) of the aqueous humor was collected at specific time points (1, 4, 6, 24, 48, 96, and 192 h) by inserting a 30 G needle into the anterior chamber at 9 o’clock position on limbus. The BRI concentration was detected by a triple-quadrupole mass spectrometer (TSQ Quantum ULTRA, Thermo, Waltham, MA) with an ESI probe with a liquid chromatograph (LC-20AD, Shimadzu, Kyoto, Japan). Chromatographic separation was achieved on a Hypersil Gold Dim-C18 reversed phase column (100 mm × 2.1 mm, 5 μm) with a column temperature of 40 °C. A mixture solution of 0.1% formic acid–acetonitrile was used as flow phase with a binary gradient of 10% in 0–2 min, 10–90% in 2–3 min, 90% in 3–4.5 min, and 90–100% in 4.5–4.6 min at a flow rate of 0.3 mL/min. The mass spectrometer was operated in the positive electron spray ionization mode, and quantification was performed using multiple reaction monitoring of the transitions from *m/z* 292.1 → *m/z* 212.0 for the compound. The collision energy was set as 31 eV and the spray voltage 3500 V. Both the vaporizer and the capillary temperatures were kept at 300 °C. Sheath and auxiliary gas pressures were 40 and 5 bar, respectively. All samples were mixed with a threefold volume of acetonitrile and centrifuged at 16,000 rpm for 10 min, and then 10 μL of the supernatant was sampled to HPLC–MS for analysis.

#### 2.5.2. IOP-lowing effectiveness

Experiments including transportation, care, and operations complied with the Association for Research in Vision and Ophthalmology Statement for the Use of Animals in Ophthalmic and Vision Research and the guidelines of the Animal Care and Use Committee of Fudan University (Shanghai, China). The IOP-lowering effectiveness of the BRI@SR@TPU implants was evaluated using New Zealand rabbits (weighing 2.5 kg, male) with normal IOP. All 12 rabbits were divided into three groups: control (*n* = 12, all left eyes), BRI eye drops (0.15%, *n* = 6), and BRI@SR@TPU implants (*n* = 6). Rabbits were anesthetized via an intramuscular injection of xylazine hydrochloride (10 mg/kg body weight), diazepam (1 mg/kg body weight), and topical anesthesia (0.5% oxybuprocaine hydrochloride). A BRI@SR@TPU implant was administrated into the cul-de-sac of rabbit right eye. The BRI@SR@TPU implant was fixed on the bulbar conjunctiva with one stitch to prevent uncontrollable scratching-off. In comparison, BRI eye drops were administrated on the rabbit cornea surface (20 μL, ×5, 10 min interval for each dose). The rabbit eyes in each group were observed using slit lamp at the time points (0, 0.5, 1, 2, 4, and 6 h, and 1, 2, 4, 6, 9, 14, 18, 21, and 28 d) after surgery to evaluate the anterior chamber. The IOP was measured at each follow-up time with an Icare^®^ TONOVET Plus rebound tonometer (Icare Finland Oy, Helsinki, Finland). The magnetic probe was kept in the horizontal position with the end of tonometer tip perpendicularly directed from 5 mm away the central cornea. An IOP value was obtained from six measurements per eye.

### 2.6. *In vivo* biosafety of BRI@SR@TPU implants

The *in vivo* biosafety of BRI@SR@TPU implants was evaluated by the anterior segment photographs and histopathological analysis of eye tissues. After 28-days follow-up, all rabbit eyeballs were enucleated for histopathological examination. The eyeballs were fixed in 4% paraformaldehyde for 48 h and then embedded in paraffin. Sections (5 µm) were cut and stained with hematoxylin and eosin (H&E).

### 2.7. Statistical analysis

The results were expressed as mean ± SD. Statistical analysis was conducted using SPSS 13.0 (SPSS Inc., Chicago, IL). One-way ANOVA test was performed to compare different groups. Statistical differences were regarded as significant when *p*< 0.05.

## 3. Results and discussion

### 3.1. Preparation and characterization of BRI@SR@TPU implants

**Figure 2.**
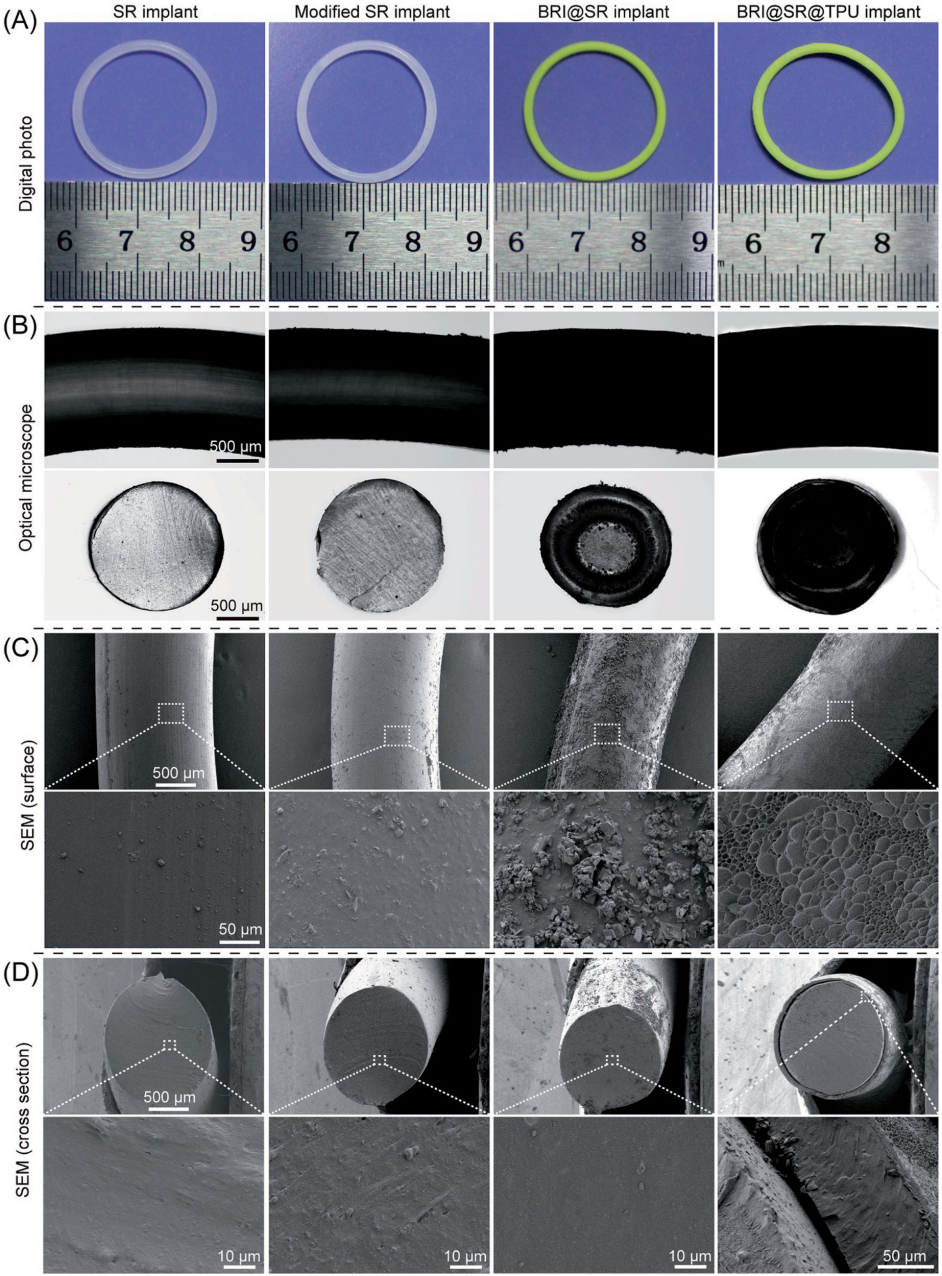
Macroscopic and microscopic morphologies of the SR implants before and after the modifications through NaOH, APTES, and tartaric acid, loaded with brimonidine (BRI) and coated with thermoplastic polyurethanes (TPUs). (A) Digital photos; (B) optical microscopy photos; (C) outside surface photos and (D) cross-sectional photos of the SR implants, modified SR implants, BRI@SR implants, and BRI@SR@TPU implants observed by SEM.

The SR implants had an average mass of 135.55 ± 2.86 mg, average volume of 141.69 ± 8.31 mm^3^, average density of 0.96 ± 0.04 g/cm^3^, with an average cross-sectional diameter of 1.66 ± 0.04 mm. After the SR implants were modified through NaOH, APTES, and tartaric acid, their average mass, volume and density changed to 108.85 ± 2.42 mg (by 19.70%), 125.72 ± 6.84 mm^3^ (by 11.27%), and 0.86 ± 0.04 g/cm^3^ (by 10.42%), and their average cross-sectional diameter changed to 1.53 ± 0.04 mm (by 7.83%). The macroscopic and microscopic morphologies of the SR implants before and after the modifications were observed, and the results are shown in Figure 2. The SR implants had smooth surfaces and dense cross-sections with certain light transmittance (column 1 in Figure 2(A–D)). When the SR implants were modified through NaOH, APTES, and tartaric acid, their surfaces and cross-sections became rougher and the light transmittance decreased, which were mainly due to the formation of a large number of micropores (column 2 in Figure 2(A–D)). After loaded with BRI, most of rough microscopic pores in the cross-sections became smaller or disappeared, and the BRI@SR implants became opaque (column 3 in Figure 2(A–D)). Furthermore, the TPU coating made the coarsened surface of SR implants flattened again (column 4 in Figure 2(A–D)), and the average thickness of the TPU coating was 83.99 ± 5.14 µm.

**Figure 3.**
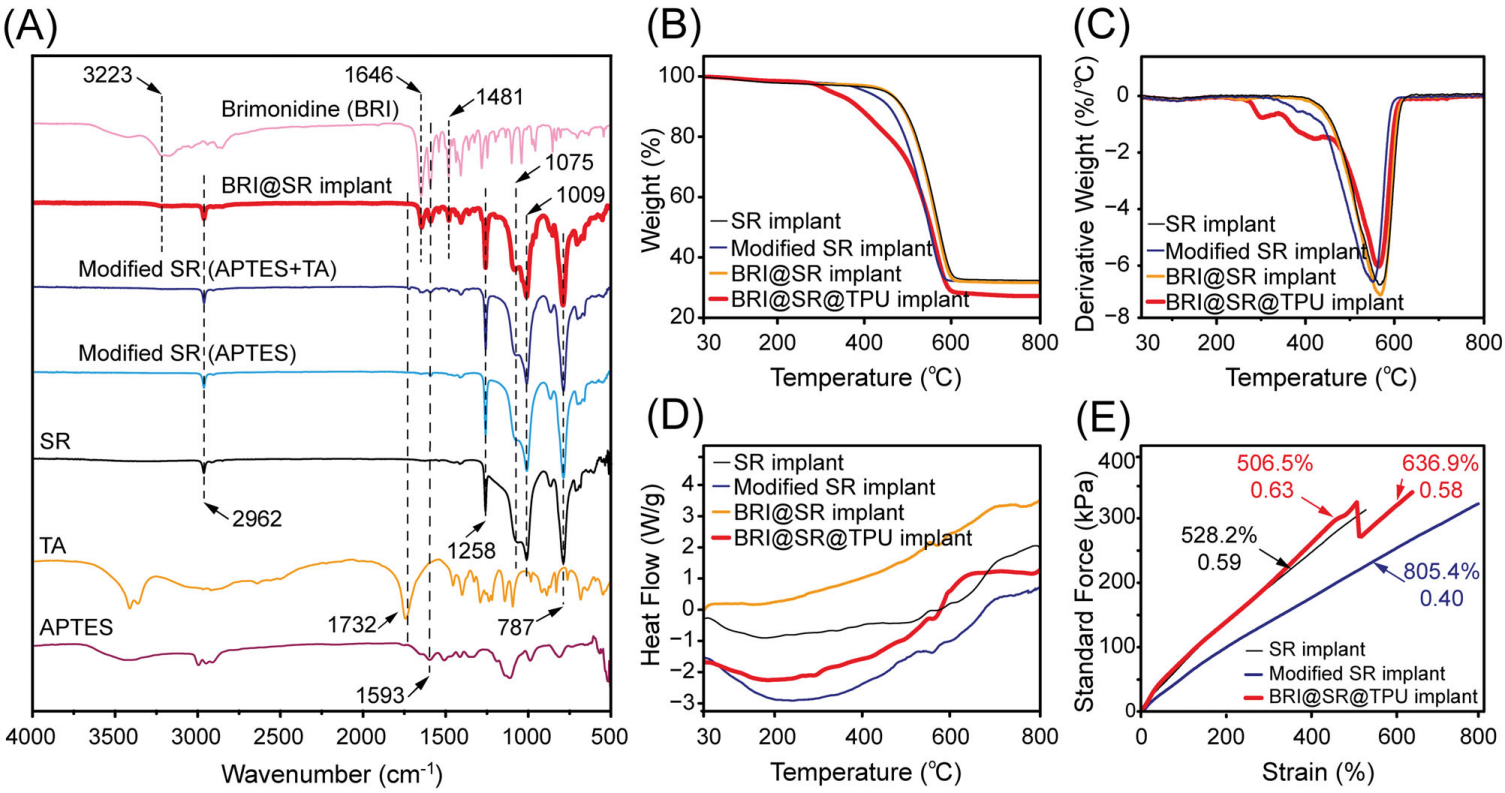
(A) FTIR spectra of APTES, tartaric acid (TA), brimonidine (BRI), the SR implants before and after the modifications with APTES and TA, and loaded with BRI; (B) thermogravimetric curves (TG) curves, (C) derivative thermogravimetric (DTG) curves and (D) differential scanning calorimeter (DSC) curves of the SR implants before and after the modifications, loaded with BRI and coated with thermoplastic polyurethanes (TPUs); (E) stress–strain tensile curves of the SR implants, modified SR implants, and BRI@SR implants.

The modifications of the SR implants and BRI-loading were investigated by FTIR, and the results are shown in Figure 3(A). The characteristic peaks of the SR implants were at 2962 cm^−1^ (CH_3_ rocking), 1258 cm^−1^ (various C–H vibrations of the SR methyl groups), 1075 cm^−1^ and 1009 cm^−1^ (Si–O–Si stretching vibration), and 787 cm^−1^ (Si–CH_3_ wagging) (Zargar et al., 2016). After the SR implants were modified with APTES and tartaric acid, the characteristic peaks of APTES (1593 cm^−1^, belonging to N–Η bending) and tartaric acid (1732 cm^−1^, belonging to C═O stretching) were observed in the FTIR curve of the modified SR implants (Figure 3(A)), which evidenced the successful modifications of the SR implants. After BRI-loading, the characteristics peaks of BRI at 1481 cm^−1^ (stretching vibration of C−C in benzene ring), 1593 cm^−1^ (N–Η bending), 1646 cm^−1^ (stretching vibration of N═C and C═C groups), and 3223 cm^−1^ (NH stretching) (Maiti et al., 2011; Sun et al., 2017), also appeared in the FTIR curve of the BRI@SR implants, which indicated BRI loaded into the modified SR implants successfully.

Thermal stability of the SR implants before and after the modifications, loaded with BRI and coated with TPU was investigated, and the TG/DTG curves are shown in Figure 3(B,C). The DTG curves contained a similar strong peak which indicated the maximum change rate of mass loss and the major pyrolysis stage of the SR implants. The SR implants had a peak of derivative weight change at 561.8 °C, while the modified SR implants had a slightly decreased peak at 550.3 °C which mainly resulted from the slightly loose microstructures when modified through NaOH, APTES, and tartaric acid (Figure 3(B)). After drug-loading, the BRI@SR implants presented an increased peak of derivative weight change at 565.1 °C, a little bit higher than the original SR implants. The peak moving-up to the high position again revealed that the BRI@SR implants had a slightly enhanced internal microstructure which was mainly due to the dense loading of BRI into plenty of internal micropores and the interaction between BRI and tartaric acid molecules grafted into the internal micropores of SR implants. Furthermore, the TPU coating on the BRI@SR implants resulted in several peaks of derivative weight change in the temperature range of 260–440 °C, which was mainly due to the thermal decomposition of the surface-coated TPU. Moreover, the DSC curves shown in Figure 3(D) presented a series of gentle exothermic peak from 560 to 570 °C which corresponded to the peaks in DTG curves and also confirmed the material compositions and mass loss of the SR implants before and after the modifications, loaded with BRI and coated with TPU. More importantly, all samples provided excellent thermal stability in the biological temperature range (∼37 °C).

As shown in Figure 3(E), the stress–strain curves of the SR implants before and after the modifications exhibited linear change, which indicated the SR implants had a perfect elasticity. A lower slope (0.40) and more strain (805.4%) were observed in the stress–strain curve of the modified SR implants compared with those of the SR implants (0.59, 528.2%), which revealed that the modified SR implants had a softer and looser microstructure. After loaded with BRI and coated with TPU, BRI@SR@TPU implants showed an elevated slope (0.63) in the strain range (<506.5%), which was similar to that of the SR implants before the modifications. Very interesting, the stress–strain curve of the BRI@SR@TPU implants presented a sharp drop of stress in the strain range of 506.5–512.0%, which mainly revealed an uneven plastic deformation of the surface-coated TPU. After a zigzag change, the elongation of BRI@SR@TPU implants further increased to 636.9% with a restored slope (0.58), which might indicate the stress–strain property of BRI@SR implants without TPU. The above results of tensile tests showed the BRI@SR@TPU implants had perfect elasticity for ease administration.

### 3.2. *In vitro* drug release from BRI@SR@TPU implants

**Figure 4.**
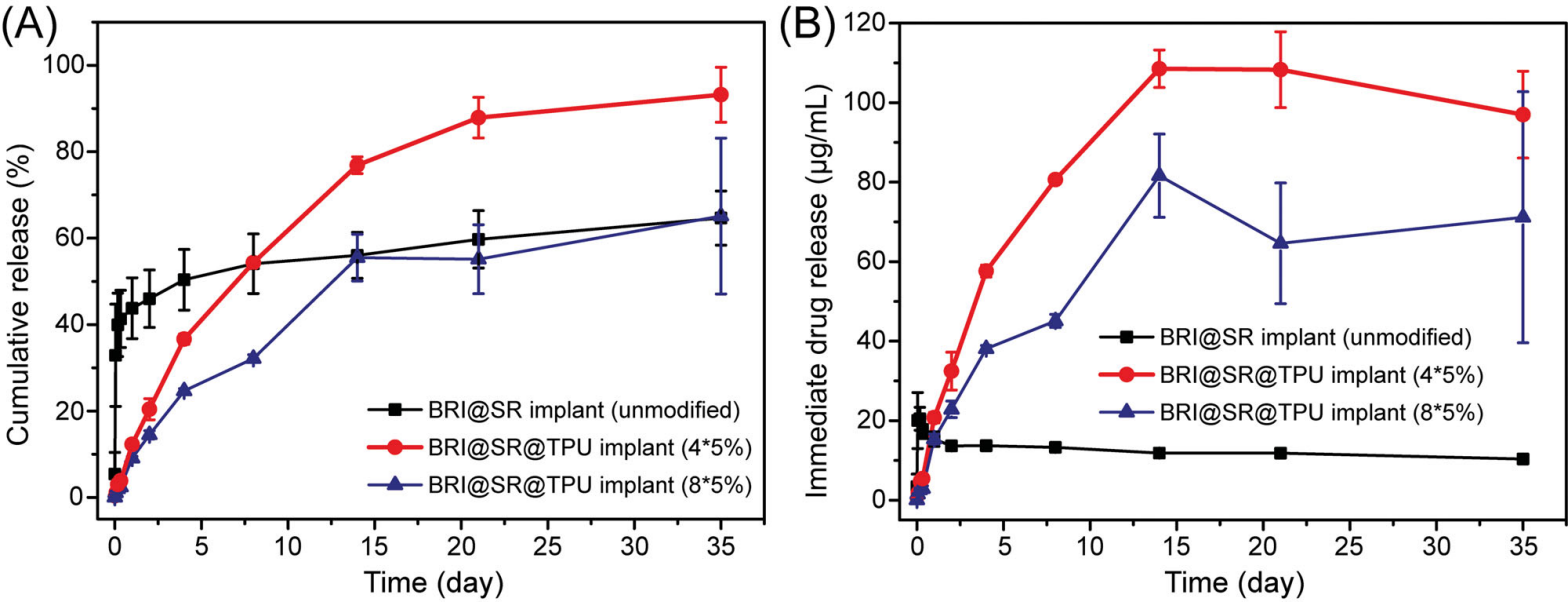
*In vitro* cumulative release profiles (A) and immediate releases profiles (B) of brimonidine (BRI) from BRI@SR implant (unmodified), BRI@SR@TPU implants (4*5%) and BRI@SR@TPU implants (8*5%).

The BRI@SR implants (unmodified) loaded BRI with an average amount of 666.7 ± 37.5 μg. As shown in Figure 4(A), the release profile of the BRI@SR implants (unmodified) experienced a significantly burst release (32.1%) in the first hour, 43.2% release in the first day, and then slow release until 35 d (62.5%), with a lower immediate drug concentration (∼12 µg/mL, Figure 4(B)). In order to improve drug loading, the SR implants were modified through NaOH, APTES, and tartaric acid to obtain a looser internal microstructure with negatively charged surface, which benefited BRI loading and releasing by electrostatic interaction. The BRI@SR implants (modified) had an average BRI loading amount of 1951.2 ± 252.9 μg, which was more than 2.9 times compared with that of the BRI@SR implants (unmodified). The drug release profile was further ameliorated by coating TPU on the surface of the BRI@SR implants. The BRI@SR@TPU implants (4*5%) provided only 1.1% release in the first hour, more than 12.3% release in the first day, and then a sustained and nearly linear release until 14 d (76.9%), followed by a slow release up to 35 d (∼100 µg/mL, 93.2%, Figure 4(A,B)). However, the BRI@SR@TPU implants (8*5%) presented 1.1% BRI release in the first hour, more than 9.1% release in the first day, and then a sustained release until 14 d (55.5%), followed by a slow release up to 35 d (∼70 µg/mL, 65.1%, Figure 4(A,B)).

The BRI@SR implants (unmodified) presented an accelerated initial burst release which was mainly due to the rich drugs loaded in the superficial layer of BRI@SR implants (unmodified) and weaker interactions with the SR carrier. The underlying mechanism was mainly associated with drug diffusion, which was similar to the previous report (Chou et al., 2016). However, the BRI@SR implants (modified) presented a more sustained drug release which was most commonly governed by a permeation mechanism involving dissolution and diffusion of drug molecules from the SR carrier with functionally modified microstructure. This was supported by the previous reports (Malcolm et al., 2016). During the 35-day observation period, the sustained-release effect of the BRI@SR@TPU implants (4*5%) was significantly better than that of the BRI@SR implant (unmodified) in terms of the decreased burst release, slower and sustained drug release, and higher immediate drug concentration. Although BRI@SR@TPU implants (8*5%) presented a little bit superiority in the decreased burst release and slower drug release compared with BRI@SR@TPU implants (4*5%), it provided lower immediate drug concentration and percentage of cumulative release. The lower percentage of cumulative release (65.1%) showed that about 35% BRI loaded in the BRI@SR@TPU implants (8*5%) could not be released during the 35-day observation period, which presented an inefficient drug utilization. Therefore, the BRI@SR@TPU implant (4*5%) was the best choice and selected to perform the following biology investigation.

### 3.3. *In vitro* cytotoxicity of BRI@SR@TPU implants

**Figure 5.**
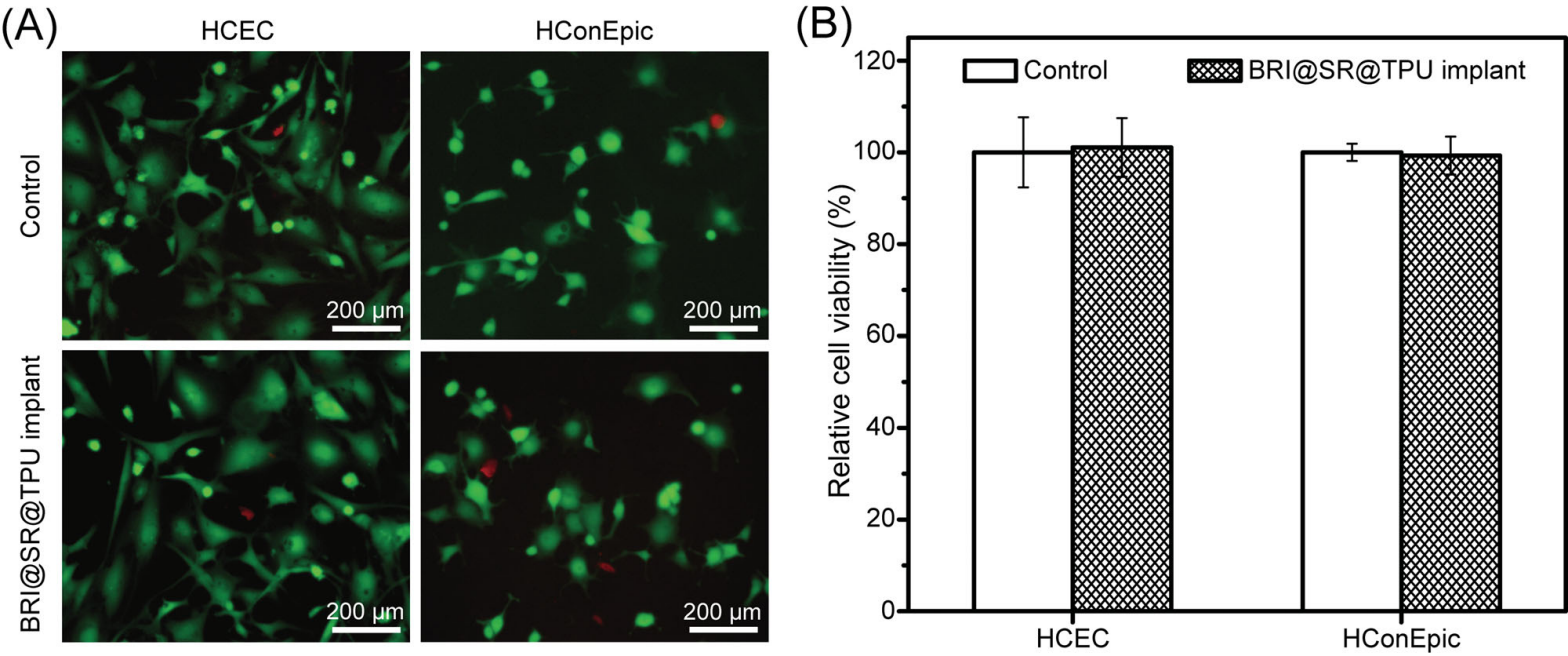
*In vitro* cytotoxicity of the BRI@SR@TPU implants on HCECs and HConEpic. The representative photos (A) and cell viability (B) of HCECs and HConEpic stained with DAPI + EDU after cells were treated with the leaching liquor of the BRI@SR@TPU implants. Cell culture wells with culture medium were set as the control (*n* = 4).

HCECs and HConEpic were used to investigate the *in vitro* cytotoxicity of the BRI@SR@TPU implants, and the representative photos are shown in Figure 5(A). The cell viability of HCECs in the BRI@SR@TPU implant group was around 101.1 ± 6.4%, when that in the control group was set as 100% (Figure 5(B)). There was no significant difference between the two groups, which demonstrated that the SR and TPU had no *in vitro* cytotoxicity to HCECs. Moreover, the results of HConEpic cells also confirmed the ignorable cytotoxicity of the BRI@SR@TPU implants, in which the cell viability was 99.3 ± 4.1%. The above results demonstrated that the BRI@SR@TPU implants had excellent biosafety, which could be used for the *in vivo* administration.

### 3.4. *In vivo* BRI release and sustained IOP-lowing effectiveness

**Figure 6.**
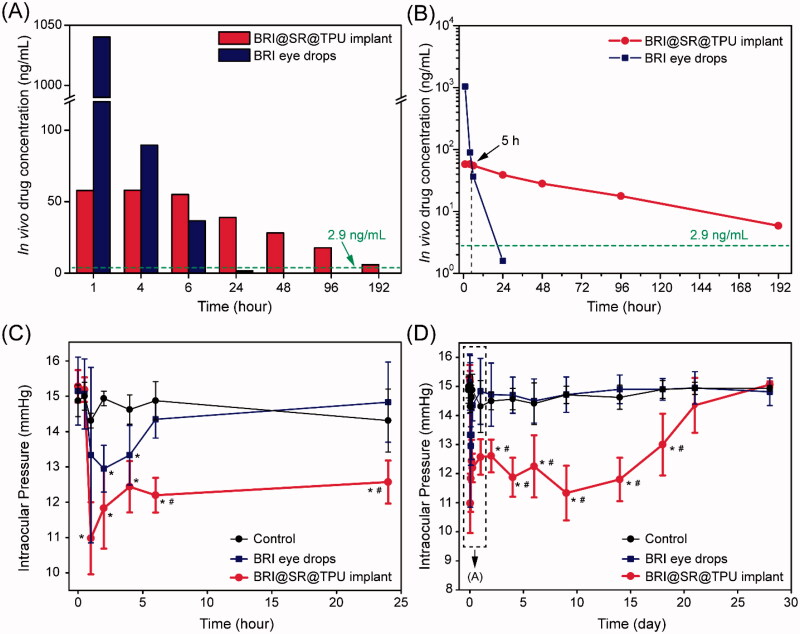
The histograms (A) and downtrend curves (B) of the *in vivo* brimonidine (BRI) concentration in the aqueous humor after administration of the BRI@SR@TPU implants or BRI eye drops. In order to improve detection accuracy, four aqueous humor samples were combined, and then dried by lyophilization. All samples were dissolved again by 50% methanol before detection by HPLC–MS. Short-term (C) and long-term (D) IOP-lowering profiles of rabbit eyes in the control (blank), BRI eye drop, and BRI@SR@TPU implant groups (*n* = 6). *^,#^Significant difference at the *p*< 0.05 level to the control and BRI eye drops at each time site, respectively.

The *in vivo* drug release was investigated by detecting the BRI concentration in the aqueous humor after administration of BRI eye drops (0.15%) or BRI@SR@TPU implants. As shown in Figure 6(A), the highest drug concentration (1040.2 ng/mL) was observed within 1 h in the BRI eye drop group, thereafter a rapid decline of BRI concentration until 24 h. In comparison, the BRI concentration in the BRI@SR@TPU implant group kept a relatively high level (58.0 ng/mL) within 4 h, which was significantly lower than that in the BRI eye drop group. The ‘burst release’ of BRI when using BRI eye drops was obviously inhibited in the BRI@SR@TPU implant group, which might relax the possible side effects caused by the ‘burst release’ of BRI. After the critical time point (about 5 h), the BRI@SR@TPU implant provided a relatively stable and slowly descending BRI concentration at 6 h (55.0 ng/mL) and 24 h (39.0 ng/mL), much higher than those in the BRI eye drop group (36.6 ng/mL at 6 h, and 1.6 ng/mL at 24 h, Figure 6(B)). After 48 h, no enough BRI was detected in the aqueous humor in the BRI eye drop group, while a considerable BRI concentration (28.1–5.9 ng/mL) was still maintained in the BRI@SR@TPU implant group. It should be noted that the above BRI concentration profile in the BRI eye drop group was in good agreement with that obtained by the fluorometric and radioactivity measurements (Acheampong et al., 1995). It was also reported that the required BRI level in the aqueous humor was 2.9 ng/mL (i.e. 0.01 μM) according to the EC50 (concentration for 50% of maximal effect) for the functional activity of BRI as α2-adrenergic agonist (Acheampong et al., 1995). Thus, the improved *in vivo* drug release profile would significantly prolong the drug action time and enhance the treatment of glaucoma. Whether eye drops or drug implants, it was inevitable that part of the drugs dissolved in the tear film entered systemic circulation by conjunctiva absorption (Hosoya et al., 2005). Fortunately, the BRI@SR@TPU implant significantly increased ocular absorption and inhibited systemic absorption when compared with the BRI eye drops, which was supported by the previous report (Pang et al., 2018).

Treatment effectiveness of BRI@SR@TPU implants was evaluated by investigating the IOP-lowering effect. The IOP decreased markedly in 1–2 h in both BRI eye drop (0.15%) and BRI@SR@TPU implant groups compared to the control group in which no significant IOP-lowering effect was observed (Figure 6(C)). Then, IOP restored in the BRI eye drop and BRI@SR@TPU implant groups, and finally to the platform level which was similar to that in the control group (Figure 6(D)). Compared with the BRI eye drop group (only 6 h), the BRI@SR@TPU implant significantly reduced IOP and sustainably maintained lower IOP until day 18, which was partly supported by the *in vivo* BRI concentration in the aqueous humor (Figure 6(A,B)). These data demonstrated that the BRI@SR@TPU implant extended the IOP-lowering effect for a much longer period (18 d vs. 6 h, 72 times), showing a potential alternative for effective treatment of glaucoma.

As we all know, the experimentally induced glaucomatous animal model is a better choice to evaluate the IOP-lowering effectiveness of DDSs (Lai & Hsieh, 2012). For anti-glaucoma drugs that reduce IOP through a pressure-dependent (trabecular meshwork) pathway, such as pilocarpine, glaucomatous animal model with elevated IOP is the best choice. However, the anti-glaucoma drug used in this paper, BRI, is a highly selective α2-adrenergic agonist which reduces IOP by reducing aqueous humor production and increasing aqueous humor outflow via the uveoscleral pathway which is pressure-independent (Adkins & Balfour, 1998). The animal model with normotensive eyes has been demonstrated to present a significant IOP lowering effect after an administration of BRI DDS (Sun et al., 2017). Therefore, to initially investigate the IOP-lowering effect of the BRI@SR@TPU implant and exclude any uncontrollable interference, we used a relatively simple animal model with normal IOP.

### 3.5. *In vivo* biosafety of BRI@SR@TPU implants

**Figure 7.**
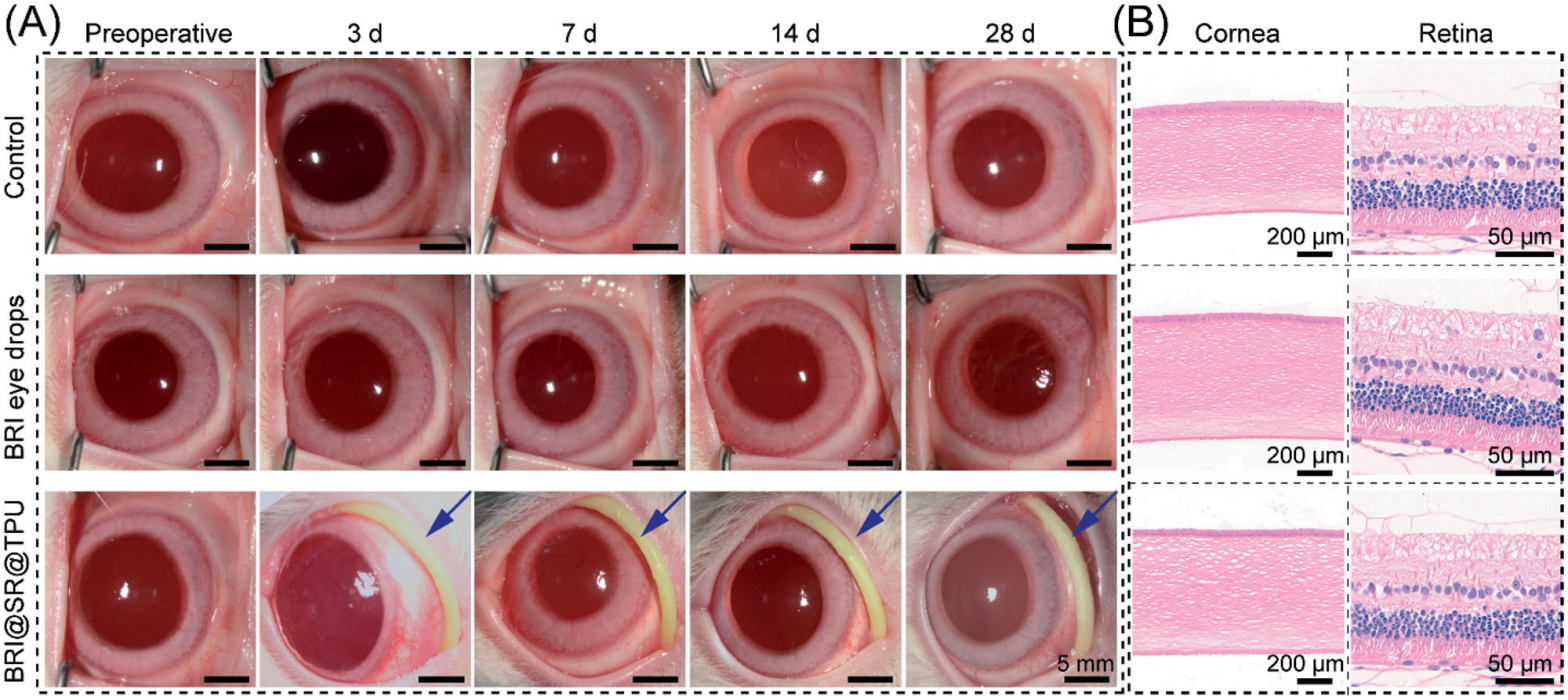
(A) Representative photos of ocular anterior segment preoperative and at day 3, 7, 14, and 28 after an administration of brimonidine (BRI) eye drops (0.15 wt%) or the BRI@SR@TPU implant which was marked by a blue arrow (row 3). In order to observe the sample more clearly, the rabbit eyelids were pulled open carefully when the photos were taken. (B) Representative photos of histological structures of cornea and retina stained with H&E after 28 days of observation.

The *in vivo* biosafety of the BRI@SR@TPU implants was evaluated by observing and comparing the representative photographs of ocular anterior segment and ocular histological structures in the BRI@SR@TPU implant, BRI eye drop and control groups. As shown in Figure 7(A), no apparent abnormity was observed after administration of BRI@SR@TPU implant or BRI eye drops. Specifically, only tiny amount of non-purulent secretion was observed in the BRI@SR@TPU implant group at day 3 and 7 post-administration, which might be due to the mild congestion of local conjunctiva tissues by sutures. Moreover, no obvious difference/abnormality of tissues and cell morphology were found in the pathological photos of the eye histological structures (including cornea and retina) in the BRI@SR@TPU implant group compared to the BRI eye drop and control groups (Figure 7(B)). These findings suggested that the BRI@SR@TPU implants had no distinct tissue toxicity, and could be used for ocular disease treatments by conjunctival sac administration.

## 4. Conclusions

A highly efficient BRI@SR@TPU implant for sustained release of BRI was designed, prepared and characterized in this study for sustained relief of intraocular pressure to treat glaucoma. The *in vitro* BRI release from the BRI@SR@TPU implant showed a sustainable release up to 35 d, with decreased burst release and increased immediate drug concentration. The BRI@SR@TPU implants were not cytotoxic to human corneal epithelial cells and conjunctival epithelial cells, and showed good biocompatibility for safe administration into rabbit’s conjunctival sac. The BRI@SR@TPU implants presented sustained *in vivo* BRI delivery and effectively relieved intraocular pressure for 18 day compared to the commercial BRI eye drops (6 h). The BRI@SR@TPU implant is thus a promising noninvasive treatment platform for long-term IOP reduction in patients with glaucoma and ocular hypertension.
